# Couples Adjusting to Multimorbidity: A Dyadic Study on Disclosure and Adjustment Disorder Symptoms

**DOI:** 10.3389/fpsyg.2019.02499

**Published:** 2019-11-08

**Authors:** Andrea B. Horn, Victoria S. Boettcher, Barbara M. Holzer, Klarissa Siebenhuener, Andreas Maercker, Edouard Battegay, Lukas Zimmerli

**Affiliations:** ^1^University Research Priority Program “Dynamics of Healthy Aging”, University of Zurich, Zurich, Switzerland; ^2^Center of Competence Multimorbidity, University of Zurich, Zurich, Switzerland; ^3^Psychopathology and Clinical Intervention, Department of Psychology, University of Zurich, Zurich, Switzerland; ^4^Department of Clinical Psychology and Psychotherapy, Bielefeld University, Bielefeld, Germany; ^5^Department of Internal Medicine, University Hospital Zurich, Zurich, Switzerland; ^6^Department of Internal Medicine, Cantonal Hospital Olten, Olten, Switzerland

**Keywords:** interpersonal emotion regulation, disclosure, complex health situations, multimorbidity, adjustment disorder symptoms, preoccupation, failure to adapt, sleep problems

## Abstract

**Background:**

Multimorbidity is challenging not only for the patient but also for the romantic partner. Strategies for interpersonal emotion regulation like disclosing to the partner are supposed to play a major role in the psychosocial adjustment to multimorbidity. Research has often focused on disease-related disclosure, even though disclosing thoughts and feelings related to mundane, everyday life occurrences might also play a role in coadjustment. The current dyadic study aimed at investigating the association between these two types of interpersonal regulation strategies and adjustment disorder symptoms, following the new ICD 11 criteria in multimorbid patients and their partners.

**Methods:**

Shortly after being hospitalized due to an acute health crisis, *N* = 28 multimorbid patients (average age 70 years) and their partners filled in questionnaires on disclosure in the couple, adjustment disorder criteria of the ICD 11 (“preoccupation,” “failure to adapt”), and sleep problems.

**Results:**

Both patients and their partners did show similarly high levels of preoccupation and failure to adapt indicating adjustment problems to the complex health situation. The adjustment symptoms of both partners correlated between *r* = 0.22 and 0.45. Regression based on Actor-Partner Interdependence-Models revealed that own mundane disclosure was related to less adjustment symptoms in the patients. Beyond that, a partner effect was observed, revealing a negative association between partners’ illness-related disclosure and the patients’ level of preoccupation. For the partners, mundane disclosure of the partner was associated with less preoccupation, failure to adapt, and reported sleep problems above and beyond own disclosure reports. Furthermore, there was an actor effect of disease-related disclosure on less sleep problems for the partners.

**Conclusion:**

These results support an interpersonal view on adjustment processes to physical disease. Disclosure as a way of regulating the relationship and emotional responses might play a relevant role here, which seems to be different for patients and their partners. Further research is needed to shed more light on the differential role of disease-related and mundane everyday disclosure for psychosocial adjustment in couples confronted with health challenges.

## Introduction

Multimorbidity—commonly defined as the co-occurrence of two or more chronic conditions—is the most frequent disease pattern in the adult population in high-income countries (Fortin et al., 2012; Tinetti et al., 2012). Multimorbidity increases with age, with prevalence estimates about 50% and more for people 65+. Health care costs (Lehnert et al., 2011) and the individual burden of multimorbidity (Fortin et al., 2004; Vancampfort et al., 2017) increase with every single additional disease (McPhail, 2016). Moreover, the social context of the patient is profoundly affected and might play an often underestimated role in the adjustment processes of the patient. Within the social context, the closest relationship in adulthood is the romantic partner. It is therefore to expect that the partner has a central role in the adjustment to medical incidences and in the context of multimorbidity where long-term management of multiple diseases is one of the greatest health-related challenges patients face.

In this study, we investigate the process of adjustment to an acute health crisis in the context of multimorbidity. To be more specific, the role of disease-related and everyday disclosure as a predictor of stress response will be examined. We follow a socio-interpersonal perspective on adjustment symptoms as a stress response in the context of multimorbidity (Schulze et al., 2014).

The interpersonal perspective on coping processes when adjusting to morbidity has yielded increasing support in the field (Berg and Upchurch, 2007; Kayser et al., 2007; Helgeson et al., 2018; Rentscher, 2019). Many of these views refer to interpersonal ways of coping as “communal coping.” In many theoretical propositions, the importance of shared appraisals regarding the situation has been underlined (Helgeson et al., 2018; Rentscher, 2019), such as a construal of the disease as *we* disease. This is in line with suggestions by other researchers pointing to the importance of the perception of the disease as a shared, yet commonly manageable, problem (Kayser et al., 2007). Besides the activation of individual resources like self-efficacy and improved self-regulation as pathways related to communal coping in disease situations, relationship quality has been suggested as a genuinely relational mechanism (Helgeson et al., 2018). Similarly, recent research on emotion regulation leaves the earlier adopted “lone man against the elements” view on regulating mood and emotion behind and underlines the role of genuinely interpersonal coregulating processes. Again, relationship quality has been shown to be a key candidate for an interpersonal pathway of emotion regulation in the literature (Debrot et al., 2013; Horn et al., 2018). Emotion regulation refers to processes that involve the activation of a goal to change, strengthen, or decrease emotional experiences and is thus a broader concept than coping as it includes the cultivation and maintenance of positive states without any demanding situations that need to be coped with (Gross, 2013). Emotion regulation has risen more and more interest in the literature as it is at the core of affective well-being (Aldao et al., 2010) and health (DeSteno et al., 2013). Thus, the idea of coregulation of emotions *via* a good relationship with the partner to recover from negative emotional “downs” and maintaining positive states might explain why the mere presence of a romantic partner is so healthy (Coan and Sbarra, 2015; Kiecolt-Glaser and Wilson, 2017) and the subjective feeling of being lonely is so dangerous (Selcuk and Ong, 2013; Holt-Lunstad et al., 2015; Slatcher and Selcuk, 2017). But what do we know about the establishment of positive relationship quality? A prominent model explaining this is the process model of psychological intimacy; it postulates an interactive process involving disclosure of personal relevant information of one interaction partner as a start point. When disclosure is followed by a responsive reaction, it leads to a shared notion of being close, understood, and validated, which in turn constitutes relationship quality (Reis and Shaver, 1988).

In Rimé’s research of social sharing after an emotionally difficult situation, the mere process of disclosing or sharing the upsetting experience is seen as an interpersonal emotion regulation strategy as it nurtures basic socio-affective needs (Rime, 2007). In the context of cancer, the literature reveals strong evidence of the importance of disclosure and resulting psychological intimacy between romantic partners when adjusting to the disease (Manne et al., 2004, 2018; Manne and Badr, 2008). These findings have been replicated in the management of other diseases like arthritis (Zhaoyang et al., 2018) and have informed influential theories in the field underlining the importance of disclosure in the context of illness adjustment (Manne et al., 2004; Lepore and Revenson, 2007). When including both patient and partner and applying dyadic analysis, the findings have been mixed: some speak in favor of a primarily intrapersonal effect of disclosure (“I feel better, when I open up”) in contrast to interpersonal or partner effects (“My partner feels better when I disclose”); it seems that there can be “too much” disclosure, particularly if the partner has more need to share concerns than the patient, a phenomenon linked to depressive symptoms (Hagedoorn et al., 2011). Theoretically, interpersonal effects are to be expected and might sometimes might not been detected due to methodological problems (like shared method variance within the same person’s self-reports of self- and partner disclosure). Moreover, interindividual differences might explain the mixed findings in the field. As an example, different attachment styles could explain why different kinds of disclosure sometimes are more or less helpful depending on the interpersonal needs related to different attachment styles (Vilchinsky et al., 2010, 2015; Pietromonaco et al., 2013). Furthermore, the literature suggests gender differences in coping with stressful experiences; interpersonal strategies, also referred to as “tend-and-befriend,” have been seen as more female than the supposedly male “fight-or-flight”-related coping repertoire (Taylor, 2006).

Most studies on the role of disclosure for disease management have focused on illness-related content. As an exception, there is a study showing that relationship-related disclosure is beneficial for couples coping with lung cancer (Badr et al., 2008). These results are in line with the idea that in real life, the coregulation of emotional states even in difficult situations might depend less on specific support situations or sharing of deeply personal content but rather on ordinary interactions in daily life. That is what the Relational Regulation Theory (RRT) posits (Lakey and Orehek, 2011). Evidence in support of the RRT shows that the positive effects of social support are linked to mental health due to mundane social interactions and the resources that develop as a result of the interactions. A recent study relying on audio sensing in daily life of breast cancer patients and their partners also showed that couples even during treatment do not talk a lot about cancer and that daily life-related everyday disclosure was associated more closely with positive adjustment (Robbins et al., 2018).

To sum up, conceptually and empirically informed, there are reasons to believe that both the sharing or disclosure of disease-related thoughts and concerns and everyday life disclosure regarding mundane experiences are related to positive mental health outcome when adjusting to an illness. As outlined above, one could argue that illness-related disclosure has the potential to foster shared appraisals of the disease in the couple, leading to more successful communal coping. It furthermore should foster psychological intimacy and thus relationship quality as it involves opening up about personal thoughts and feelings. However, it might have downsides regarding emotional contagion (Bolger et al., 1989) and mismatch in needs (Hagedoorn et al., 2011). In contrast, mundane everyday life disclosure should not help establish shared appraisals of the disease but maintain a positive relationship quality in a more unspecific way as it also involves sharing of positive content. RRT would suggest that it offers a social regulation resource and has less risk for difficult situations as they have been reported in the context of social support. In the literature, it could be shown that being the support receiver has downsides that might be explained with threats to autonomy and self-esteem (Bolger and Amarel, 2007; Maisel and Gable, 2009; Zee and Bolger, 2019).

Failed adjustment to illness as a mental health problem is a well-established finding, for example, in individuals with cancer (Mehnert et al., 2013), cardiac surgery (Oxman et al., 1994), and other health problems (Foster and Oxman, 1994). Multimorbidity itself can be seen as a risk factor for mental health problems In a representative Scottish sample of over a million patients in primary care, patients with a diagnosis of depression—a diagnosis that can be interpreted as one form of chronic adjustment problems—were most likely multimorbid (Smith et al., 2014). From a mental health perspective, it has often been criticized that the diagnosis of adjustment disorder, even though broadly used in clinical practice, lacks a conceptual framework (Strain and Diefenbacher, 2008). In the upcoming ICD 11, new criteria will be introduced for adjustment disorder symptoms that are informed by a stress response perspective on adjustment disorder (Maercker et al., 2013). A stress-response framework allows bridging findings and concepts from stress and trauma research to the field of adjustment disorder (Maercker et al., 2007). Accordingly, it has been proposed that all kinds of stress responses not only after trauma but also after critical life events should be seen in their social context. The socio-interpersonal model of stress response, as proposed by Maercker and Horn (2013), has been supported by a couple of studies considering the socio-interpersonal context of stress response (Krutolewitsch et al., 2016; Lorenz et al., 2018b).

Adjustment disorder in the ICD 11 is defined as an “emotional disturbance arising as a consequence of a significant life event” (Maercker et al., 2013, p. 381). In contrast to trauma as an etiological requisite of posttraumatic stress disorders (PTSDs), the stressor is not supposed to be outside the realm of usual human experience. The acute distress reactions around the stressful event, however, may be just as strong as to that of a trauma. In the context of PTSD, these reactions are called peritraumatic distress reactions (Brunet et al., 2001) and represent well-established risk factors for long-term symptoms (Thomas et al., 2012). The stress responses to the significant life event are grouped into two symptom clusters. The first symptom cluster represents a maladaptive reaction to the identifiable psychological stressor that leads to psycho-social functional impairment and is referred to as the symptom group “failure to adapt.” The second cluster is characterized by preoccupation with the stressor and its concomitants (Glaesmer et al., 2015). Sleeping problems are an important indicator of failure to adapt to the stressor (Lorenz et al., 2018a). In the context of physical health conditions, this is of particular interest as sleep disruptions might be a pathway bridging socio-affective phenomena with metabolic reactions associated with physical health (Kiecolt-Glaser and Wilson, 2017). First big-scale studies relying on this diagnostic system of adjustment disorder are promising (Glaesmer et al., 2015; Zelviene et al., 2017; Ben-Ezra et al., 2018; Maercker and Lorenz, 2018). It has been proposed that particularly older multimorbid patients are prone to adjustment disorder as the conditions are requiring heavy effort when it comes to instrumentally (i.e., regarding medical routines) and emotionally (considering the chronic perspective of the condition) adjusting to the multitude of diagnosis and their consequences in the patient’s daily life (Schulze et al., 2014). However, so far, to our knowledge, no study has investigated adjustment problems to multimorbidity or even other physical health problems following the state-of-the-art framework of ICD 11.

This study aims at investigating the adjustment of couples facing an acute health crisis in the context of multimorbidity and the role of disclosure. To our knowledge, this is the first study presenting data of adjustment disorder within the new ICD 11 framework not only of the patient but also of the partner in the context of physical health problems. To foster further research on possible risk factors for adjustment disorder in medical contexts, clinically relevant adjustment problems in both partners will be presented in relation to indicators of multimorbidity-related impairment and peri-admission distress. In particular, we will investigate patient and partner effects of everyday life and disease-related disclosure on preoccupation and failure to adapt, the core symptoms of adjustment disorder as defined in the ICD 11, and sleep problems. Investigating both disease-related and everyday life disclosure allows as to distinguish the expected differential effect of both disclosure types. Based on the literature outlined above, we expect both kinds of disclosure to be useful. However, following RRT (Lakey and Orehek, 2011), everyday life disclosure is expected to be more important for general regulatory responses to the stressful situation. It can also be seen as having less possible downsides like a mismatch of sharing needs (Hagedoorn et al., 2011) as compared to disease-related disclosure and thus be more efficient in activating relational resources. Therefore, we expect everyday life disclosure to have more pronounced effects on adjustment problems.

## Materials and Methods

### Procedure

This study is part of a project named GUGKS (Gemeinsamer Umgang mit gesundheitlich komplexen Situationen–Couples Coping With Multiple Chronic Medical Conditions). Eligible for study participation were inpatients at the Department of Internal Medicine of the University Hospital of Zurich. Inclusion criteria were minimum 18 years of age, multimorbidity (i.e., at least two chronic conditions), having a romantic partner, language proficiency, and informed consent of both partners. Exclusion criteria were dementia, addiction, pregnancy, palliative situations, or participation in another research study within the last 4 weeks before inclusion.

Between July 2013 and April 2017, inpatients at the Department of Internal Medicine were screened by clinical staff who provided study information to eligible patients and their romantic partners as well as contact to the study team. After both partners were fully informed about the study and consented to participation, the study procedures included filling in paper-pencil questionnaires independently for both partners and a couple interview. At all points, research assistants provided support and answered questions. Relevant clinical data and reason of admission were taken from the electronic health chart. Data were pseudonymized, and the Cumulative Illness Rating Scale (CIRS, see measures) was rated by a medical doctor and calculated for each patient. This study was carried out in accordance with the recommendations of the Human Research Act of Switzerland with written informed consent from all participants. All participants gave written informed consent in accordance with the Declaration of Helsinki. The study protocol was approved by the local ethics committee (KEK-ZH-Nr.: 2013-0009).

### Participants

A total of 644 patients were screened, of which 129 did not meet the inclusion criteria. A total of 352 explicitly declined to participate (mostly arguing with the current burden of the health situation), and 135 did not participate for other reasons (e.g., patients have been transferred to other departments or have already been discharged). In total, 28 couples participated in the study; *N* = 11 female and *N* = 17 male patients and their heterosexual partners (Table 1). Among partners, there was one partner not reporting adjustment symptoms but all other variables and another only not reporting sleep problems. Therefore, in the regression models, *N* = 27 were included. Mean age of the patients was *M* = 70.22 and for their partners *M* = 68.5, with a range from 47 to 90 years (age range: patients 47–89 years, partners 55–90 years). They were married from 3 to 65 years (*M* = 28.91, *SD* = 16.55). Most participants were retired (*N* = 22 patients, *N* = 19 partners) and had middle/higher vocational training (*N* = 12 patients, *N* = 13 partners) or a college/university degree (*N* = 14 patients, *N* = 10 partners). Patients were diagnosed with a range of 3–11 chronic conditions (*M* = 6.67 conditions, *SD* = 2.07). All chronic conditions diagnosed in these patients are listed in Table 2. Mean *CIRS* expressing the burden of multimorbidity of a patient was 17.11 (*SD* = 5.28).

**TABLE 1:** Patient characteristics.

	**Number**	***SD***
**Gender**		
Male	17	
Female	11	
Age in years, mean	70.22	(10.8)
**Education**		
Basic vocational training	2	
Middle/higher vocational training	12	
College/university degree	14	
**Professional status**		
Retired	19	
Disability pension	3	
Employed	6	
Number of chronic diseases, mean	6.67	(2.07)
CIRS, mean	17.11	(5.28)
**Reason for hospital admission**		
Unspecified, fever, pain etc.	10	
Cardiovascular	4	
Respiratory	4	
Blood, blood forming organs, immune mechanism	3	
Musculoskeletal	2	
Others	5	

**SD*, standard deviation*; CIRS*, Cummulative Illness Rating Scale.*

**TABLE 2:** Multiple chronic conditions of the patients participating in the study.

**Chronic condition**	**Frequency**
Atrial fibrillation	9
Cancer (e.g., prostate, breast, skin, colon)	8
Cardiovascular disease	7
Cerebrovascular disease	4
Chronic kidney disease	18
Chronic obstructive pulmonary disease (COPD)	3
Depression	3
Diabetes	4
Digestive disease (e.g., obesity)	5
Ear disease (e.g., presbyacusis)	3
Endocrine disorders (e.g., thyroid dysfunction)	7
Epilepsy	1
Eye disease (e.g., retinopathy)	4
Gout	1
Hematological disease (e.g., anemia)	9
Heart failure	2
Hypertension	16
Ischemic heart disease	9
Lipid disorders	7
Liver disease	8
Respiratory disease	3
Musculoskeletal disease (e.g., chronic back pain, osteoarthritis)	20
Neurological disorders (e.g., peripheral neuropathy)	6
Parkinson’s disease	1
Peptic ulcer disease	5
Peripheral vascular disease	4
Prostate problems	5
Pulmonary embolism	3
Skin disease (e.g., chronic ulcer skin, seborrheic dermatitis)	9
Valvular heart disease	3

### Measures

In the following, all measures used will be presented.

#### Adjustment Disorder New Module

The Adjustment Disorder New Module (ADNM) has been developed in the context of stress response model adjustment disorder (Einsle et al., 2010) and represents the state-of-the-art scale to screen adjustment disorder symptoms (Zelviene et al., 2017; Ben-Ezra et al., 2018; Lorenz et al., 2018a). In this study, two scales of the main symptom groups of the ADNM-19 version were used (Ben-Ezra et al., 2018). The two main symptom groups are maladjustment (three items; example item: “Since the stressful situation, I find it difficult to concentrate on certain things.”) and preoccupation (four items; example item: “I have to think about the stressful situation a lot and this is a great burden on me.”).

For the assessment of the two scales, participants indicated on a 4-point Likert scale, ranging from 1 (never) to 4 (often), how they experienced symptoms during the past 2 weeks. All symptoms were assessed as a response to the stressful event of the patient being admitted to the hospital due to acute health problems. Furthermore, a short screening scale, ADNM-4, was calculated by summing up two items of each scale (Ben-Ezra et al., 2018), with a cut-off value of 8.5 suggesting a clinically significant level of adjustment symptoms.

#### Disclosure

Everyday life disclosure was assessed with nine items. The patients were asked whether they talked in daily life to their partner in the months before the hospital admission about beautiful and bad things that happened in daily life, how they felt, what bothered or moved them, what they liked and did not like about the relationship, about what made them happy, and what provoked thought (see Appendix Table A1 for details).

Illness-related disclosure was assessed with five items: it was assessed whether since the hospital admission patients and their partners talked about the patients’ current health status, own thoughts, feelings, and concerns about the patients’ health, as well as medical information regarding the health situation. All items had a 5-point Likert scale ranging from “never/not at all applicable (1)” to “multiple times a day/totally applicable (5)”; the scales represent the mean value of the item scores.

The mundane disclosure scale showed a good internal consistency (both partner and patient version Cronbach α = 0.88). The illness-related disclosure scale performed satisfying internal consistency in the patient version Cronbach’s α = 0.85, for the partner version Cronbach’s Alpha was with α = 0.56 marginally consistent.

#### Jenkins Sleep Scale

The scale was designed as an efficient and brief questionnaire to assess frequency and intensity of sleep difficulties (Jenkins et al., 1988). Four items address difficulties falling asleep, awakenings during the night, trouble maintaining sleep, and feelings of fatigue or sleepiness despite receiving typical night’s rest. The answer options range from “1 = not at all,” “2 = 1–3 days per month” to “5 = 15–21 days per month” and “6 = 22–31 days per month” and build a global mean score. The original scale had internal consistency ratings ranging from Cronbach’s Alpha 0.63–0.79. In this sample, the patient version Cronbach’s Alpha was α = 0.75 the partner version, α = 0.89.

According to sleep disorder criteria of 15 (DSM IV) or 12 days per month (DSM 5), the report of at least one sleep problem for 15 or more days has been interpreted as an indicator of clinically relevant sleep problems (Lallukka et al., 2011).

#### Other Measures

##### Peri-admission distress

To assess distress provoked by the circumstances of the hospital admission, we used four modified items from the Peritraumatic Distress Questionnaire (PDQ) (Brunet et al., 2001) to assess peri-admission distress. The PDQ was originally designed to measure the PTSD criterion A2 in the DSM IV. Patients and their romantic partners were asked whether they had experienced the following signs of distress as a response to the acute situation leading to hospital admission of the patient: fear that they/their partner would die; helplessness; shame about their affective reaction; whether they felt that they lost control over their emotions. Reliability analyses revealed that the item “I felt ashamed over my emotional reaction” was not internally consistent with the scale. Thus, a total scale was derived with the remaining three items, yielding satisfactory Cronbach alphas of α = 0.69 (patients) and α = 0.79 (partners).

##### Cumulative illness rating scale

The CIRS is a weighted sum score of the coexisting medical conditions in a patient categorized by organ systems. Every medical condition is assigned to one of the 14 defined organ domains and rated according its medical severity from 0 to 4 (Linn et al., 1968; Hudon et al., 2007).

The purpose of CIRS is to provide an index of total chronic medical illness burden and a measure of multimorbidity (Fortin et al., 2005). In lay-person language adjusted, modified versions, we asked patients (Patient-CIRS) and their romantic partners (Partner-CIRS) to report their perceived impairment in the corresponding organ domains.

### Analytical Strategy

To assess interdependencies within the couples (patient, partner) regarding being over the in the literature suggested cut-off for clinically significant adjustment disorder symptoms (Ben-Ezra et al., 2018) or not, Chi squared analyses within a 2 × 2 cross-tab were conducted. Mean differences of illness severity indicators and peri-admission distress between “over cut-off vs. not” groups were tested with ANOVAs.

In order to take the covariation of disease-related and everyday disclosure into account, a modified version of regression-based Actor Partner Interdependence Models (APIM) was conducted, following the regression-based approach presented by Kenny et al. (2006) and illustrated by Bodenmann and Ledermann (2008), which seemed an adequate strategy for the given low sample size. Two separate multiple regressions were conducted for patient and partner for each adjustment symptom group (preoccupation, failure to adapt, sleep problems) serving as dependent variable. Predictors are own and partner every day and disease-related disclosure. Interdependencies between the predictors and residuals of both regression models are reported as a Pearson correlation coefficient. An essential prerequisite for multiple regressions is a normal distribution of the residuals (Li et al., 2012). To test this, Shapiro–Wilk test was applied. Following recent recommendations, we report the *p*-values, effect sizes (standardized coefficients *r* and beta) and do not rely on the term “statistically significant” but rather include the confidence interval in the interpretation (Hurlbert et al., 2019).

## Results

In Table 3, all study variables’ mean and SD as well as correlations within individuals are depicted. Regarding the patients’ data, the independent variable everyday disclosure correlated with a medium effect size and *p*-values of *p* = 0.001 and *p* = 0.04 with the dependent variables preoccupation and failure to adapt. Smaller correlations were present between dependent and independent variables regarding partner data. The calculation of patient-partner correlations revealed several medium-sized correlations between dependent and independent variables, which are displayed in Table 4. Besides disclosure types, preoccupation shows high associations between partners in the couples. The bivariate correlations also show that patients’ everyday disclosure is associated with less symptoms in the partner.

**TABLE 3:** Means, SDs, and correlations of disclosure and adjustment problems (patient-patient and partner-partner).

	**1**	**2**	**3**	**4**	**5**
1 Illness-related disclosure	1	0.44^∗^	0.09	0.29	0.26
2 Everyday disclosure	0.53^∗∗^	1	–0.06	–0.02	–0.09
3 ADNM preoccupation	0.02	−0.44^∗^	1	0.71^∗∗^	0.74^∗∗^
4 ADNM failure to adapt	–0.19	–0.58^∗∗^	0.62^∗∗^	1	0.71^∗∗^
5 Sleep problems	0.18	0.25	–0.01	0.07	1
Patient mean (*N*)	3.96 (28)	3.65 (28)	9.30 (27)	6.37 (27)	12.11 (28)
Patient *SD*	1.04	0.89	3.31	2.15	4.90
Partner mean (*N*)	4.22 (28)	3.60 (27)	9.74 (27)	5.11 (27)	10.96 (26)
Partner SD	0.54	0.82	3.61	2.36	5.77

**ADNM*, Adjustment Disorder New Module 19 subscales; *Sleep problems*: Jenkins Scale. Correlations: above the diagonal: partner; below the diagonal: patients. ^∗^*p* ≤ 0.05. ^∗∗^*p* ≤ 0.01.*

**TABLE 4:** Correlations patient–partner of disclosure and adjustment problems.

	**Illness-related disclosure “Patient”**	**Everyday disclosure “Patient”**	**ADNM preoccupation “Patient”**	**ADNM failureto adapt “Patient”**	**Sleep problems“Patient”**
1 Illness-related disclosure “Partner”	0.38^∗^	0.19	–0.21	–0.16	–0.09
2 Everyday disclosure “Partner”	0.54^∗∗^	0.45^∗^	0.11	–0.22	–0.19
3 ADNM preoccupation “Partner”	–0.31	–0.56^∗∗^	0.44^∗^	0.46^∗^	–0.25
4 ADNM “failure to adapt “Partner”	–0.26	−0.45^∗^	0.30	0.34	–0.31
5 Sleeping problems “Partner”	–0.30	–0.54^∗∗^	0.09	0.32	–0.22

**ADNM*, Adjustment Disorder New Module 19 subscales; *Sleeping problems*: Jenkins Scale. ^∗^*p* ≤ 0.05. ^∗∗^*p* ≤ 0.01.*

### Adjustment Disorder Symptoms and Sleep Problems in Patients and Their Partners

In Table 5, the number of participants with adjustment problems in the ADNM-4 over the cut-off and clinically significant sleep problems are depicted for both patients and partners. Sleep problems seem to occur frequently but independently in the couples of this sample. In contrast, a chi-squared test suggests that (sub)clinical levels of adjustment problems were not independent between both partners [*X*^2^(1, *N* = 27) = 7.42, *p* = 0.006]; in only one couple the partner reported adjustment problems while the patient did not. All other partners did not reach the cutoff if the patient did not reach it either.

**TABLE 5:** Concurrent adjustment problems in the couple: Corresponding numbers of patients and partners with adjustment disorder symptoms (according to the ADNM4) respectively sleep problems (according to the Jenkins Scale) over the cut-off.

			**Partner**				
			**Adjustment disorder over cut-off**		**Significant sleep problems**		
			**No**	**Yes**	**No**	**Yes**	**Total**
Patient	Adjustment disorder over cutoff	No	12	1	9	5	13
		Yes	6	8	5	8	14
	Significant sleep problems	No	6	6	6	6	12
		Yes	12	3	8	7	15
		Total	18	9	14	13	

**ADNM4* cutoff: >8.5; significant sleep problems cutoff: any symptom in *Jenkins Scale* over ≥ 5. Please note that one partner report on *Jenkins Scale* and one on *ADNM 4* is missing resulting in different total *N*s.*

#### Characteristics of the Clinically Relevant Group

For heuristic purposes, the characteristics of the group with clinically relevant levels of maladjustment (adjustment disorder, sleep problems) as opposed to the non-clinical group were illustrated. Table 6 depicts different features of objective and subjective illness severity and peri-admission distress by these two groups. Descriptives and ANOVAs suggest that patients with adjustment problems are patients with higher objective (number of conditions) and subjective (Patient-CIRS) illness severity. Interestingly, even the partners’ perception of illness severity (Partner-CIRS) did play a role. In turn, partners are more likely to belong to the group over the cutoff, when patients (not doctors) reported more multimorbidity-related impairment. Furthermore, partners with clinical levels of adjustment disorder reported stronger distress around the hospital admission of their partners.

**TABLE 6:** Means and SDs of indicators of illness severity and peri-admission distress by groups “over cutoff” yes versus no.

	**CIRS total (medical doctor)**		**Total number of chronical conditions**		**CIRS total (patient)**		**CIRS total (partner)**		**Peri-admission distress (patient)**		**Peri-admission distress (partner)**	
	***M (SD)***	***F***	***M (SD)***	***F***	***M (SD)***	***F***	***M (SD)***	***F***	***M (SD)***	***F***	***M (SD)***	***F***
ADNM 4 patient ≥ 8.5												
No	11.55 (4.39)		5.18 (1.89)		8.14 (4.02)		11.15 (5.13)		2.29 (1.18)		1.98 (1.00)	
Yes	15.58 (6.78)		7.33 (2.57)		14.86 (6.57)		16.29 (9.39)		2.77 (0.80)		2.38 (1.08)	
Total	13.65 (6.00)	2.82	6.30 (2.48)	5.15^∗^	11.50 (6.35)	10.64^∗∗^	13.81 (7.94)	3.03’	2.53 (1.02)	1.64	2.18 (1.04)	1.06
ADNM 4 partner ≥ 8.5												
No	12.21 (4.96)		6.36 (2.90)		10.94 (7.17)		11.82 (6.63)		2.42 (1.08)		1.81 (0.89)	
Yes	16.50 (7.27)		6.25 (1.91)		13.11 (4.54)		17.89 (9.44)		2.74 (0.97)		2.81 (1.07)	
Total	13.77 (6.11)	2.71	6.32 (2.53)	0.01	11.67 (6.40)	0.68	13.92 (8.08)	3.67’	2.52 (1.04)	0.57	2.15 (1.05)	6.66^∗^
Patient sleep problems ≥ 5												
No	15.00 (6.86)		6.44 (2.51)		10.17 (5.95)		14.00 (7.52)		2.22 (0.56)		2.36 (1.03)	
Yes	12.79 (5.47)		6.21 (2.55)		12.50 (6.63)		13.67 (8.52)		2.76 (1.23)		2.04 (1.06)	
Total	13.65 (6.00)	0.74	6.30 (2.48)	0.05	11.50 (6.35)	0.92	13.81 (7.94)	0.01	2.53 (1.02)	1.98	2.18 (1.04)	0.64
Partner sleep problems ≥ 5												
No	12.64 (5.32)		5.82 (2.71)		8.93 (4.07)		11.71 (6.46)		2.29 (0.90)		2.10 (0.90)	
Yes	14.73 (6.96)		6.27 (1.62)		13.54 (7.28)		17.33 (8.11)		2.79 (1.14)		2.33 (1.21)	
Total	13.68 (6.14)	0.63	6.05 (2.19)	0.23	11.15 (6.18)	4.21^∗^	14.31 (7.67)	3.87’	2.53 (1.04)	1.66	2.21 (1.05)	0.34

**ADNM 4*, Adjustment Disorder New Module 4. *Sleep problems ≥ 5*: at least one symptom as assessed in Jenkins sleep scale over 5, that is, clinical cutoff; *CIRS*, Cumulative Illness Rating Scale assessed by doctor/patient/partner; *F* values comparing means above and below the cut-off scores. ’*p* ≤ 0.10. ^∗^*p* ≤ 0.05. ^∗∗^*p* ≤ 0.01.*

### APIM Analyses on Adjustment Disorder Symptoms and Sleep Problems

Regression-based APIMs were used to investigate whether the independent variables everyday disclosure and illness-related disclosure of patients and partners predicted preoccupation, failure to adapt, and sleep problems of both parties. All results are gathered in Table 7. The Shapiro–Wilk test suggested normally distributed residuals in all presented regression analyses.

**TABLE 7:** Regressions to predict “failure to adapt,” preoccupation, and sleep problems of the patient via everyday disclosure and illness-related disclosure of patient and partner.

	**Patient failure to adapt**			**Patient preoccupation**			**Patient sleep problems**		
	**β**	***t***	**95% CI**	**β**	***t***	**95% CI**	**B**	***t***	**95% CI**
Patient (Actor Effect)									
Everyday disclosure	–0.71^∗∗^	–3.305	[−2.745 – −0.625]	–0.75^∗∗^	–3.767	[−4.473 – −1.291]	0.11	0.473	[−2.234 – 3.552]
Illness-related disclosure	0.31	1.316	[−0.342 – 1.521]	0.43’	1.979	[−0.068 – 2.729]	0.39	1.508	[−0.692 – 4.384]
Partner (Partner Effect)									
Everyday disclosure	<0.01	0.004	[−1.157 – 1.161]	0.40’	1.910	[−0.142 – 3.337]	–0.41	–1.659	[−5.563 – 0.618]
Illness-related disclosure	–0.14	–0.716	[−2.031 – 0.991]	−0.41^∗^	–2.220	[−4.688 – −0.153]	–0.09	–0.392	[−4.887 – 3.334]
*R*^2^	0.38			0.46			0.18		
*F*	3.18^∗^			4.51^∗∗^			1.20		

	**Partner failure to adapt**			**Partner preoccupation**			**Partner sleep problems**		
	**β**	***T***	**95% CI**	**β**	***t***	**95% CI**	**B**	***t***	**95% CI**

Partner (Actor Effect)									
Everyday disclosure	0.15	0.703	[−0.871 – 1.761]	0.21	0.969	[−1.114 – 3.060]	0.12	0.572	[−2.315 – 4.063]
Illness-related disclosure	0.37′	1.940	[−0.113 – 3.260]	0.16	0.816	[−1.625 – 3.723]	0.41^∗^	2.267	[0.353 – 8.467]
Patient (Partner Effect)									
Everyday disclosure	−0.58^∗^	–2.691	[−2.803 – −0.359]	–0.64^∗∗^	–2.876	[−4.616 – -0.742]	−0.57^∗^	–2.74	[−6.605 – −0.895]
Illness-related disclosure	–0.14	–0.627	[−1.396 – 0.749]	–0.08	–0.347	[−1.984 – 1.416]	–0.17	–0.777	[−3.441 – 1.574]
*R*^2^	0.43			0.38			0.46		
*F*	3.92^∗^			3.25^∗^			4.26^∗^		

*‘*p* ≤ 0.1. ^∗^*p* ≤ 0.05. ^∗∗^*p* ≤ 0.01. *95% CI*, 95% confidence intervals of the unstandardized Betas. Actor Effects (Patient on Patient; Partner on Partner) are displayed on top, followed by Partner Effects underneath (Patient on Partner, Partner on Patient).*

With regard to the dependent variable failure to adapt, it was found that the independent variables explained 37.7% of the variance for patient symptoms and 42.8% of partners’ failure to adapt. Patient’s everyday disclosure was negatively related to the patient’s score on failure to adapt, which depicts an actor effect. The 95% confidence interval of unstandardized *Betas* suggests with a probability of 95% a −0.625 up to −2.745 lower value in the outcome when a 1 point higher score is reported on the 5-point Likert scale of disclosure. Simultaneously, patient’s everyday disclosure predicted lower failure to adapt scores by the partner. This means that a partner effect of patients’ everyday disclosure is present; here, the 95% confidence interval suggests with 95% chance a −0.359 to 2.803 lower value per one unit more reported on the disclosure 5-point Likert scale. The residuals of these models correlated with *r* = 0.23 (*p* = 0.28). The higher the correlation of the residuals, the higher is the non-independence of relevant third variables not considered in the model. A factor shared in the environment of both partners, which is not considered in the model, should lead to high correlations of the residuals. A relevant, though not considered, factor that is disease-related and only relevant for the patient, but not the romantic partner, should lead to low correlations of the residuals.

APIM regression models with the dependent variable preoccupation explained 46.2% of the variance in patients and 38.2% in their partners. A higher extent of patients’ everyday disclosure and partners’ illness-related disclosure predicted lower patients’ preoccupation scores with standardized betas suggesting small to medium effect sizes and p values between 0.001 and 0.038. Confidence intervals of the unstandardized betas are depicted in Table 7. Results suggest an actor effect of everyday disclosure as well as a partner effect of illness-related disclosure. Relating to the dependent variable patients’ sleep problems, the independent variables explained 17.9%. Increasing patient’s everyday disclosure was associated with less preoccupation of the partner. Thus, again, a partner effect for patients’ everyday disclosure was observed with a standardized beta of −0.64, suggesting a medium effect size. The residuals of these two models correlated with *r* = 0.28 (*p* = 0.18), suggesting some covariation within the couples between relevant variables not considered in the models.

Lastly, none of the independent variables predicted sleep problems in the patient with a *p*-value below 0.05 (see Table 7 for complete data), while in their partners, 46.0% of the variance of the dependent variable sleep problems was explained by the independent variables. Partners’ sleep problems were related to more own illness-related disclosure and less everyday disclosure of the patient. Results suggest an actor effect for the illness-related disclosure and a partner effect for the everyday disclosure. Residuals correlated with *r* = −0.07 (*p* = 0.75), which suggests different relevant variables outside the model for patients and their romantic partners.

## Discussion

This study aimed at investigating adjustment to an acute health crisis in the context of multimorbidity from a socio-interpersonal perspective, including the patients and their partners. The occurrence of adjustment symptoms in the ICD 11 in patients and their partners was investigated. Furthermore, actor and partner effects of everyday life and disease-related disclosure on adjustment were examined to answer the questions whether (1) disclosure plays the expected protecting role against adjustment symptoms, and (2) whether there are distinguishable effects of everyday and illness-related disclosure.

### Prevalence of Adjustment Symptoms

As a first step, the prevalence and interdependence of adjustment symptoms in patients and their partners were investigated. When relying on a screening tool (Ben-Ezra et al., 2018) to assess indicators of clinically significant adjustment problems, results underline the high prevalence of adjustment and sleep problems in our sample in both patients and their partners. Our data suggest a prevalence similarly high in significant (often caregiving) others as in the patients themselves and can be interpreted as a call for considering romantic partners in further investigations of adjustment disorder in the medical context. For adjustment disorder, an association within the couple was found—when the patient reported critically elevated levels of adjustment disorder symptoms, there is a higher risk for the romantic partner to be affected as well. Bivariate correlations reveal a continuous relationship only in the symptom group “preoccupation” between partners, suggesting that excessive worrying seems to spill over to the partner. Those patients who reported adjustment problems tend to have more diagnoses, perceive their impairment as more pronounced (Patient-CIRS), and tend to have partners who perceive the impairment as high (Partner-CIRS). It is interesting to note that patients’ and partners’ perception of illness severity seems to be more predictive for adjustment problems than the medical doctors’ evaluations. This is in line with the literature on illness perception (Leventhal et al., 1997) showing evidence that subjective evaluations of the illness situation—not only by the patient but also by the patients’ partners (Karademas and Giannousi, 2013)—are predictive for adjustment (Hagger and Orbell, 2003). In accordance with findings from trauma research (Brunet et al., 2001), peri-admission distress by the partner was associated with a more severe stress response syndrome. Together with earlier findings showing adverse effects of partner distress in illness adjustment (Rohrbaugh et al., 2009), this suggests the importance of partners’ distress around patients’ acute crises and has implications for optimizing procedures considering the needs of relatives, for example, in the context of clinic admissions.

### Actor and Partner Effects of Different Types of Disclosure on Adjustment

Addressing the research question whether higher levels of disclosure are associated with better adjustment, APIMs revealed distinct pattern between everyday- and illness-related disclosure in patients and their partners. No actor effects of patients’ illness-related disclosure on own or partner symptoms could be observed. In contrast, the extent of patients’ everyday disclosure before the admission was highly related to less adjustment symptoms in the symptom groups for preoccupation and failure to adapt. Standardized betas of 0.71 and 0.75 suggest considerable effect sizes of this association. Moreover, partner effects of patients’ reported every day disclosure on their partners’ adjustment symptoms and sleep problems were observable with relatively high effect sizes (betas between 0.57 and 0.64) on all three symptom groups. However, the partners’ mundane exchanges with the patient were not predictive for adjustment; everyday disclosure did not show any significant relationships with own or patient’s preoccupation, failure to adapt, or sleep problems. In contrast, partners’ illness-related disclosure showed an opposite pattern of actor and partner effects; it was associated with more own sleep problems and less preoccupation in patients. The latter adaptive association for patients’ outcomes is in line with earlier findings (Robbins et al., 2014). Bivariate correlation hint in the same directions, making suppression effects leading to artifacts in the regression models unlikely. In general, the observed effects on sleep quality are particularly interesting as sleep disturbances in response to interpersonal processes have been identified as representing a pathway of psychosocial events to physical health through its known associations to metabolic syndrome, depression, and inflammatory processes (Kiecolt-Glaser and Wilson, 2017). Thus, identifying psychosocial factors associated with sleep problems means identifying possible pathways representing important body-mind links, which in turn has implications for interventions.

To sum up, patient’s everyday disclosure, the amount of sharing thoughts and feelings regarding positive and negative mundane and relationship-related experiences assessed in retrospect, was in this sample associated with less symptoms not only on own adjustment symptoms but also on all symptom groups of their partners. This can be seen as in line with RRT (Lakey and Orehek, 2011) and could be interpreted as highlighting the importance of processes underlying the maintenance of positive relationship quality within the acute health situation. This interpretation corresponds to other studies indicating meaningful relationships between processes related to relationship quality (responsiveness) and sleep quality (Selcuk et al., 2017) as well as other physical (Robles et al., 2014) and mental health (Whisman, 2007) outcomes. Furthermore, the observed effects of everyday disclosure could be a result of the inclusion of positive content. Sharing positive experiences in couples is named capitalization, which is a strong predictor of positive relationship quality. Capitalization allows to capitalize the benefit of positive experiences by sharing them and fostering positive social exchange processes with the partner that have been associated with a number of positive relationship outcomes (Peters et al., 2018). In general, even substantive conversations as opposed to small talk have been shown to be related to life satisfaction in daily life—a finding that has been replicated in big ecologically valid samplings in real life (Milek et al., 2018). This seems in line with our findings, maintaining contact by exchanging daily content is associated with less distress.

Does everyday disclosure of patient buffer them and their partners from possible downsides of the patient and support receiver role by maintaining social exchange that functions beyond the patient-caregiver roles? Does the maintenance of mundanity correspond to the needs of maintaining autonomy and independence that often has been reported in the context of chronic disease (Eckerblad et al., 2015)? Does it help to maintain dignity and buffers against feelings of being a burden to the caregiver—aspects that have been identified as crucial for keeping up the will to live in severely ill patients near the end of life (Chochinov et al., 2005)? Does, in turn, the improved relationship quality and mental health of the patient also serve as protective for the partners’ distress reactions on the complex health situation with all of the demanding implications between caregiving and living with a constant health threat? Are female gender and presence of chronic disease in caregivers associated with a higher prevalence of adjustment disorder (Loh et al., 2017)? Further research is needed to address these questions that seem worthwhile to follow up when looking at the results of this small, but highly burdened, sample. When focusing on the differential effects of everyday life disclosure, it is interesting to note that while both adjustment disorder symptoms preoccupation and failure to adapt seem to be associated, the patient’s sleep quality did not show any association with both kinds of own and partner disclosure. In this sample, possibly this is due to a ceiling effect in sleep quality due to the acute health crisis and the hospital situation. Further research is needed to explore whether this would replicate in other patient samples with less acute health situations.

In contrast, illness-related disclosure did show less consistent associations. First, partners’ but not patients’ illness-related disclosure was relevant for the observed outcome in this sample. Second, partner who talked more about the thoughts and feelings regarding the disease reportedmore preoccupation and worse sleep quality. At the same time, however, this was associated with less preoccupation in the patient. One could argue that individuals who are very preoccupied with the health situation of their partners have more urge to share their repetitive thoughts and feelings. This phenomenon is referred to as co-rumination in the literature (Rose et al., 2007) where it has been shown as maladaptive when adjusting to stressors (Horn and Maercker, 2016). In adolescence, co-rumination is a risk factor for depression onset but at the same time it has found to be associated with better friendship quality (Rose et al., 2007). Possibly, a similar effect was observable in our sample. Disease-related disclosure was associated with a positive outcome in the patient, which can be seen as a result of a potential improvement of relationship quality due to more open conversations. This is also in line with earlier findings pointing to the importance of the partner’s need to talk about the disease for adjustment (Hagedoorn et al., 2011); it might be an indicator of a need for further interventions when the partner has an urge to talk a lot about his or her concerns related to the health situation of the patient.

In another line of arguing, illness-related disclosure as a way of updating shared illness appraisal (Helgeson et al., 2018) was possibly less important in our sample, where all patients have had suffered from chronic conditions for a longer time period. Possibly, the establishment of shared appraisal as a pathway of communal coping of the medical situation might be more important in earlier stages of more recently diagnosed diseases. Moreover, the term “social constraints” has been elaborated in the literature referring to the lack of opportunities to disclose also disease-related content to others due to negative reactions by the social context (Cordova et al., 2001; Herzer et al., 2006; Braitman et al., 2008; Agustsdottir et al., 2010; Pasipanodya et al., 2012). In contrast to mundane content, disease-related thoughts and feelings might be more at risk to provoke social constraints, an expectation that should be investigated in further research.

### Implications and Outlook

The reported findings and the results of this study foster the discussion about future interventions for couples coping with multimorbidity as it is already in discussion in the field of cancer (Badr, 2017) or stress in general (Lavner and Bradbury, 2017). There is rising evidence pointing to the importance of offering space and encouraging mundane ways of interacting and talking to the partner. This opens the opportunity to activate interpersonal resources and coregulate difficult situations in the context of demanding medical conditions. It furthermore has direct relational consequences that in turn strengthen the couple to cope better and have a better shared understanding of the medical situations. The improvement of relationship quality in turn opens resources needed for instrumental and emotional support by the partner. Moreover, it strengthens the sense of autonomy and independence, which are perceived as threatened in challenging health situations (Eckerblad et al., 2015). It has been suggested that adjustment disorder symptoms are particularly suited for e-health interventions (Kocalevent et al., 2015). Even though this recommendation was based on individual counseling, current e- and m-health developments also offer the possibility to provide couple-based e-interventions. That might be a promising path for further development in the field.

In another stream of reasoning, disclosure with the partner might be seen as a field to explore own health goals. Particularly in the context of multiple chronic conditions, patient preferences and disease treatment guidelines tend to conflict with each other. Studies involving stakeholder perspective (Ferris et al., 2018) and leading multimorbidity experts in the field (Tinetti et al., 2016) vote for a patient-centered rather than disease-centered treatment approach. It is, however, not a trivial task for a multimorbid patient to weigh the pros and cons of certain treatment decisions. Exchanging with close others like the romantic partner is an important support to find words for own preferences and values that later can be shared with a medical treatment team (Naik et al., 2018). Including the partner in this process seems particularly worthwhile. First, the close relationship might allow the security of allowing to express own concerns and emotional reactions. Second, the partner most times is involved in the caregiving and thus some decisions might be linked to the capacities of the caregiver. Unfortunately, current interventions on patient-centered care for multimorbid patients do not commonly include the relatives in their approaches (Poitras et al., 2018). It is, however, important to keep in mind that there sometimes might be conflicts between needs of autonomy and communal coping and shared appraisals. Complex health situations are often correlated with high levels of dependency on caregivers, an experience often related to perceived loss of autonomy and associated with feelings of guilt toward the partner (Eckerblad et al., 2015). Future interventions need to be tailored to the individual relationship styles of the patients and their partners as it is represented in the attachment style (Vilchinsky et al., 2010, 2015; Pietromonaco et al., 2013). Furthermore, they should consider the possible conflict between needs for autonomy and communal coping. From the partners’ perspective, it needs to be taken into account whether and how the partner’s role is defined as a caregiver (Cipolletta et al., 2013) as well as the cultural background (Parveen et al., 2011).

### Limitations

There are several limitations to be considered to avoid premature conclusions. A first and important limitation is the small sample size. Research with bigger sample sizes is crucial. The explained variance and effects sizes were relatively high in the regression analyses, however, clearly a bigger sample is needed to replicate the findings.

Second, the sample is possibly selected as a high number of eligible couples did not will to participate in the study. On an anecdotal note, most patients and their partners appreciated the acknowledgment of the complexity of the situation within an acute health crisis in the context of multiple chronic conditions. Particularly, romantic partners answered with gratitude that they were seen. However, many couples who did decide not to participate fed back that they felt to be too burdened by the current situation as to participate in a study. This might speak in favor of a selection of rather less burdened couples in our sample. Considering that not only half of the patients but also half of the partners even in this sample showed clinically relevant signs of adjustment problems, one can only guess how a representative sample in a similar acute situation might be burdened. As screening measures are not at hand that are not burdening to patients or relatives, representative studies seem warranted to get an insight about the actual prevalence of adjustment disorder in own or the partners’ acute health crises.

Third, this study is dyadic, allowing to assess partner effects that are free of shared method variance related to response sets, which seems an advantage. However, all disclosure measures rely on retrospective self-reports with all their limitations (Bolger et al., 2003; Tennen et al., 2006). Furthermore, it cannot be excluded that in the assessment of general negative disclosure in the everyday life disclosure scale, the couples also referred to illness-related disclosure. Further research with clearer separated measures and including observations of conversations in the lab (Hagedoorn et al., 2011) or even better, in daily life (Robbins et al., 2018), would be recommended.

Furthermore, this is a cross-sectional study; all associations cannot be interpreted as causal. Also, the temporal direction of the effect— is better adjustment an antecedent of everyday disclosure or does disclosure precede better mental health–cannot be investigated within this study. Further longitudinal research is needed to shed light on the temporal unfolding and causal directions of the observed effects.

## Conclusion

With all its limitations, this study opens the door for further research on couple processes in the context of multimorbidity and chronic diseases. In acute complex health situations, not only patients but also their partners seem highly challenged in their adjustment, often to a clinically relevant extent. Results speak in favor of the importance of the sharing of mundane, everyday life experiences, daily ups and downs, besides thoughts and feelings regarding the health situation. They furthermore suggest distinguishable roles for patients and their partners when it comes to the correlates of disclosing within the relationship. Considering the interdependencies between patient’s and partner’s adjustment symptoms and the importance of genuinely relational processes like disclosure, the results of this study call for integrating significant others in the treatment of multimorbidity.

## Data Availability Statement

The datasets generated for this study are available on request to the corresponding author.

## Ethics Statement

This study was carried out in accordance with current Swiss law on human research (Humanforschungsgesetz HFG) with written informed consent from patients and partners, respectively. The study protocol was approved by the local ethics committee (KEK-ZH-Nr.: 2013-0009).

## Author Contributions

AH, LZ, and BH conceived and designed the study. AH supervised the data selection and entry, and drafted the manuscript. BH organized the database. VB and AH performed the statistical analyses. All authors contributed to the writing of the manuscript and gave final approval of the version submitted.

## Conflict of Interest

The authors declare that the research was conducted in the absence of any commercial or financial relationships that could be construed as a potential conflict of interest.

## Appendix

**TABLE A1:** Questionnaire items regarding disclosure in the partnership.

Im Folgenden geht es um die **Gesprächskultur** und die **körperliche Nähe** in Ihrer Partnerschaft und den **Umgang mit Stimmungen**. Dabei gibt es **kein richtiges oder falsches Vorgehen**, jeder Mensch denkt und verhält sich in diesem Zusammenhang anders.					
**Kreuzen Sie bitte aufrichtig an, was am ehesten auf Sie und Ihren Partner zutrifft.** Die folgenden Fragen beziehen sich auf Ihr Verhalten **im vergangenen Monat** und die Reaktionen Ihres Partners darauf. Wie war das in Ihrer Partnerschaft?					
*In the following you will find a few questions regarding the* ***culture of conversation*** *and* ***physical closeness*** *in your partnership. Moreover, there are questions regarding your* ***handling of different moods***. *There is* ***no right or wrong***, *everybody thinks and acts differently in this context.* ***Please tick the answer that bests fit you and your partner.*** *The following questions refer to your behavior* ***during the last month*** *and to your partners reactions in this regard. How was it in your partnership?*					
**Im Alltag (im vergangenen Monat, bevor ich ins Spital kam) habe ich meinem Partner erzählt… *In everyday life (last month, before I was admitted to hospital) I talked to my partner about*…**					
	Trifft überhaupt nicht zu (nie) *Does not apply at all (never)*	Trifft ein wenig zu *Does apply a little bit*	Weder zutreffend noch unzutreffend *Neither accurate nor inaccurate*	Trifft eher zu *Rather correct*	Trifft voll und ganz zu (mehrmals am Tag) *Applies fully*
… was mir Schönes passiert ist.					
… *nice things that happened to me.*					
… was mir Schlechtes passiert ist.					
… *bad things that happened to me.*					
… wie ich mich fühle.					
… *how I have been feeling.*					
… was mich belastet.					
… *what has burdened me.*					
… was mich bewegt.					
… *what emotionally has touched me.*					
… was ich an ihm gut finde.					
… *what I like about him/her.*					
… was mich stört in der Partnerschaft.					
… *what has bothered me about him/her.*					
… was mich froh macht.					
… *what has made me happy.*					
… was mich beschäftigt.					
… *what has concerned me.*					

**Table:**

**Seit der Einlieferung ins Spital habe ich mit meinem Partner darüber gesprochen …**					
***Since admittance to hospital I talked to my partner about*…**					

	Trifft überhaupt nicht zu*Does not apply at all*	Trifft ein wenig zu*Does apply a little bit*	Weder zutreffend noch unzutreffend*Neither accurate nor inaccurate*	Trifft eher zu*Rather correct*	Trifft voll und ganz zu*Applies fully*

… was mich emotional bewegt, wenn ich an meine Krankheit denke.					
… *what has emotionally touched me when thinking about my illness.*					
… was ich über meine jetzige gesundheitliche Situation denke.					
… *what I have been thinking about regarding my current health situation.*					
… wenn ich etwas Neues über meine Krankheit erfahren habe (z.B. vom Arzt).					
… *any news I learned about regarding my illness (e.g. from the doctor).*					
… was mir an der jetzigen gesundheitlichen Situation Sorgen oder Angst macht.					
… *what has worried or scared me with regard to my current health situation.*					
